# Exploitation rates of two benthic resources across management regimes in central Chile: Evidence of illegal fishing in artisanal fisheries operating in open access areas

**DOI:** 10.1371/journal.pone.0180012

**Published:** 2017-06-30

**Authors:** Miguel Andreu-Cazenave, Maria Dulce Subida, Miriam Fernandez

**Affiliations:** Núcleo Milenio - Centro de Conservación Marina CCM, Estación Costera de Investigaciones Marinas, Departamento de Ecología, Facultad de Ciencias Biológicas, Pontificia Universidad Católica de Chile, Santiago, Chile; California Polytechnic State University, UNITED STATES

## Abstract

There is an urgent need to quantify the impacts of artisanal fisheries and define management practices that allow for the recovery and conservation of exploited stocks. The extent of illegal catch is particularly critical as a driver of overexploitation in artisanal fisheries. However, the lack of data at proper spatial scales limits the evaluation of illegal fishing and effectiveness of management practices. We used a catch curve analysis to estimate total instantaneous mortality as a proxy of fishing pressure in the artisanal benthic fishery in central Chile. We compared the patterns of total mortality in fishing grounds under the well-studied territorial use rights for fisheries system (TURF) immersed in a landscape of open access areas (OAA; no access restriction), and from these patterns determined the extent of illegal fishing in open access areas focusing on the two most frequently extracted resources: locos (*Concholepas concholepas*) and keyhole limpets (*Fissurella* spp.). The beauty of this seascape is the presence of the no-take (NT) area of Las Cruces as control (no fishing), allowing us to estimate natural mortality. Loco exploitation is banned in OAAs. However, loco mortality in OAAs was 92% higher than in the NT, and 42% higher than in TURFs. Keyhole limpet mortality was similar between TURFs and the NT, but doubled in OAAs. We also found strong differences in mortality among fishing grounds with the same level of protection (i.e. TURFs), and over time. Our results highlight (a) the high level of illegal fishing that may occur in artisanal fisheries under traditional management regimes, and (b) that TURFs can be effective to reduce fishing mortality. However, large variability among TURFs suggests the need for a deeper understanding of the drivers of success of TURFs.

## Introduction

Recent reports on the status of the formally assessed fisheries show that 63% of stocks are overexploited and almost half of them (45%) have reduced fishing mortality rates to levels low enough to support stock rebuilding [1]. Most of the information driving this trend, however, comes from the small fraction (20%) of the world fisheries that are assessed [2]. The remaining 80% of the world fisheries, which are unassessed, is of major concern since recent studies suggest that 64% of them are overexploited and 18% are collapsed [3]. The decline in the biomass of unassessed fisheries is a major issue [3] because they currently represent 60% of the legally reported global landings [4,5], and most of them are from small-scale, artisanal fisheries. Artisanal fisheries are critically important for diversity and food security, since almost 100% of the landings are directed to human consumption [5]. In contrast, only 57% of the landings of industrial fisheries are used as food for humans [5,6]. However, most artisanal fisheries are data-poor and formal, traditional stock assessments cannot be applied to understand and regulate fishing effort. Therefore, advances in determining the condition of unassessed stocks (and particularly small-scale fisheries), the impact of alternative management strategies, and the major drivers of overexploitation are becoming increasingly urgent.

Illegal, unregulated and unreported fishing are key factors driving fish stocks to overexploitation [7]. The most recent estimates suggest that illegal and unreported fishing range between 13 and 77% of reported catches, although large regional variability is observed [8]. Illegal and unreported fishing are particularly critical in artisanal fisheries because of the widely scattered nature of these fisheries combined with poor enforcement [8–12]. In fact, developing countries, where artisanal fisheries concentrate, are most at risk from illegal fishing [7]. In high-value species, such as abalone and lobster, illegal catch seems to be greater than legal catch in some instances [13,14]. Even in areas with high levels of enforcement, such as territorial use rights for fisheries (TURFs) illegal catches seem to be substantial [11,12,15]. In order to effectively develop and implement regulations, it is critical to first determine the extent, character and motivations of illegal fishing. Alternative methods that have been proposed for quantifying illegal fishing and its impacts include surveillance data, trade data, stock assessments based on fishery-independent (survey) data, expert opinion, and indirect observation (e.g., signs of illegal activity as an indicator of non-compliance; [7,16]). Many of these approaches, nonetheless, require monitoring as well as fisheries and fisheries-independent data. However, lack of these basic data is the common problem of artisanal fisheries. Under some scenarios, catch curve analysis offers an alternative opportunity for exploring the extent of illegal fishing as a first approach to this worldwide problem in data poor fisheries as it simply requires size (or age) frequency distribution data [17]. One scenario for this exploration is a system with contrasting levels of enforcement.

Chile offers a good scenario for exploring the potential of catch curve analysis to estimate levels of illegal fishing, and at the end assessing the effectiveness of management strategies in place. Artisanal fisheries are socially and economically important in Chile, engaging nearly 90,000 fishers [18]. The existence of two management strategies regulating the exploitation of most benthic resources for over 15 years now, allows comparisons of fishing mortality under co-management in the novel territorial use rights for fisheries system (TURF) and under traditional top-down management (e.g., bans, minimum legal size) in open access areas (OAAs). Fishing mortality can be estimated using catch curve analyses, since there is historic information on size distribution of exploited resources particularly in TURFs. Enforcement is more efficient in TURFs than in OAAs since the fishers themselves have a vested interest in protecting their TURF. Access to fishing grounds and enforcement level seem to be critical factors determining the abundance of exploited resources [19–21]. Comparative studies conducted in two management areas of central Chile showed that densities of all benthic resources and coastal fishes were higher in TURFs than in nearby OAAs [20]. Interestingly, the densities of locos (*Concholepas concholepas*), the most valuable resource, are also significantly higher in co-managed TURFs and MPAs than in open access areas [20,22], despite loco exploitation being completely banned in OAAs since 1993 [19,20,23]. This pattern of abundance suggests that illegal fishing of locos seems to occur in open access areas, and offers a platform to analyze the extent of illegal fishing in traditional, open access management regimes. To date, illegal fishing of locos has been reported inside TURFs, and it seems to be relevant [9,12,15]. Illegal fishing in open access areas may have tremendous impacts on the abundances of locos and other resources, especially considering the large fraction of the coast under an open access regime observing poor enforcement [14,24].

We conducted a snapshot study to compare the patterns of total mortality in fishing grounds under two management regimes, TURFs (named here management areas; MAs) and OAAs, and from these patterns determine the extent of illegal fishing of locos in open access areas in the central Chilean artisanal benthic fishery. We also analyzed the temporal patterns of total mortality in MA as a way to evaluate the consistency of the results of the snapshot study. We used the loco (*Concholepas concholepas*) and the keyhole limpets (*Fissurella* spp.) as model species. Since size distribution data of these resources are available for MAs, we used catch curve analysis to estimate total instantaneous mortality (*Z*) in MAs, but also in OAA. We assume that fishing mortality can be used as a proxy of fishing pressure, provided that natural mortality is known. In a coastal landscape of high fishing pressure, such as our study area in central Chile, the best estimates of natural mortality can be obtained inside marine reserves [25]. Therefore, we not only focused our work on MAs and OAAs, but also in the single no-take area found in this region. In addition to regulations on the exploitation of most benthic species (size limits, reproductive bans), loco exploitation is completely banned in OAAs. Therefore, total mortality of locos is expected to be lowest in NT and OAAs (complete ban) and higher in MAs if there is no illegal fishing. In contrast, lower total mortality of keyhole limpets is expected in the no-take (NT) area, intermediate in MAs and highest in OAAs since fishing effort has concentrated in OAAs after MAs were established. This study is of local interest as this information can be useful for local management decisions and enforcement, and also for conservation plans since MAs have been proposed as an alternative to marine protected areas, particularly in zones where human uses would not allow placing no-take areas [20,26]. This study is also of global interest as we address problems that are common in artisanal fisheries worldwide (data-poor fisheries, illegal fishing, poor enforcement), highlighting, with precautions, the value of TURFs for management and conservation.

## Material and methods

### The benthic fishery

The coast of Chile hosts one of the most productive coastal ecosystems of the world, contributing between 3 and 5% to the global catches [27; Chile ranks among the 10 top contributors to the global catch]. The artisanal benthic fishery is characterized by a TURF system that was experimentally established in the early 90s (and officially implemented in the late 90s), representing a pioneering management strategy [24,28] that is now spreading to several fisheries in Latin America and the world [5,21,29,30]. Under this TURF (MA) system, the fishers are organized within unions that administer MAs and are obligated to conduct regular stock assessments. Fishers administering a MA have exclusive fishing access in that TURF. However, the majority of the historical fishing grounds operate as open access areas [9]; only a small fraction of fishing grounds is currently under the TURF system. Along more than 800 km of coastline in the most populated region of central Chile (from 30°S to 36°S), open access fishing grounds dominate the fishing area of the benthic artisanal fisheries (on average 30% of the coastal area is assigned to MAs). In OAAs, fishers holding a fishing license can extract benthic resources following national regulations (e.g., minimum legal size, reproductive bans). The fishing licenses restrict fishing activities to regional levels (at the scale of hundreds of kilometers).

Besides small-scale comparative studies between MAs and OAAs, the status of the resources in open access fishing grounds is largely unknown [20]. Most stock assessment efforts of benthic resources are based on small-scale (TURF scale) estimates conducted by the fisher´s unions in their management areas (MAs). These assessments are generally carried out annually and provide the baseline for defining a total allowable catch in each MA, which is approved by the Undersecretary of Fisheries [19]. These patchy assessments have also been used to determine the local status of the most important resources over time [28], and to emphasize the global potential of TURFs for management and conservation [20,23]. Since the fishers have a vested interest in protecting their MAs, internal regulations foster enforcement within the unions and prevent illegal fishing by non-members. Enforcement is also patchy, being much weaker in OAAs than in MAs due to the large geographic areas at which these fisheries operate [9], the lack of compliance of fisherman with regulations in OAAs [31], and the lack of law enforcement agents capacitated in fishery issues. For these reasons, the mosaic of management strategies is linked to mosaics of enforcement and information on the status of the resource (assessment). The arbitrary fragmentation of the resources for exploitation, assessment and enforcement are major issues that hinder determining the overall status of the resources and the overall success of the management plans.

Several species are targeted by the artisanal benthic fisheries in central Chile. In OAAs a set of species including crustaceans, bivalves, gastropods, sea urchins, and others (a tunicate, several algae) are exploited. A subset of species is targeted in MAs, depending on the interests of the local fishers. However, the primary target resources in the majority of the MAs include locos (*Concholepas concholepas*), keyhole limpets (a set of *Fissurella* species) and sea urchins (*Loxechinus albus*) [23,32]. Locos can only be exploited inside MAs under a total allowable catch system (total allowable catches are also set for all target species inside the MAs); loco fishing in open access areas is prohibited [28]. MAs are fished sporadically depending on market and price opportunities, since the fisher unions independently decide on the best local strategy to harvest their annual total allowable catch. In some MAs the annual harvest may take place during a single day or few short periods totaling only a few days per year. This fact highlights an important difference between the fishing grounds under the two management regimes: that fishing occurs on a daily base in OAA, while fishing is sporadic in MA.

## Study area

**Fig 1:**
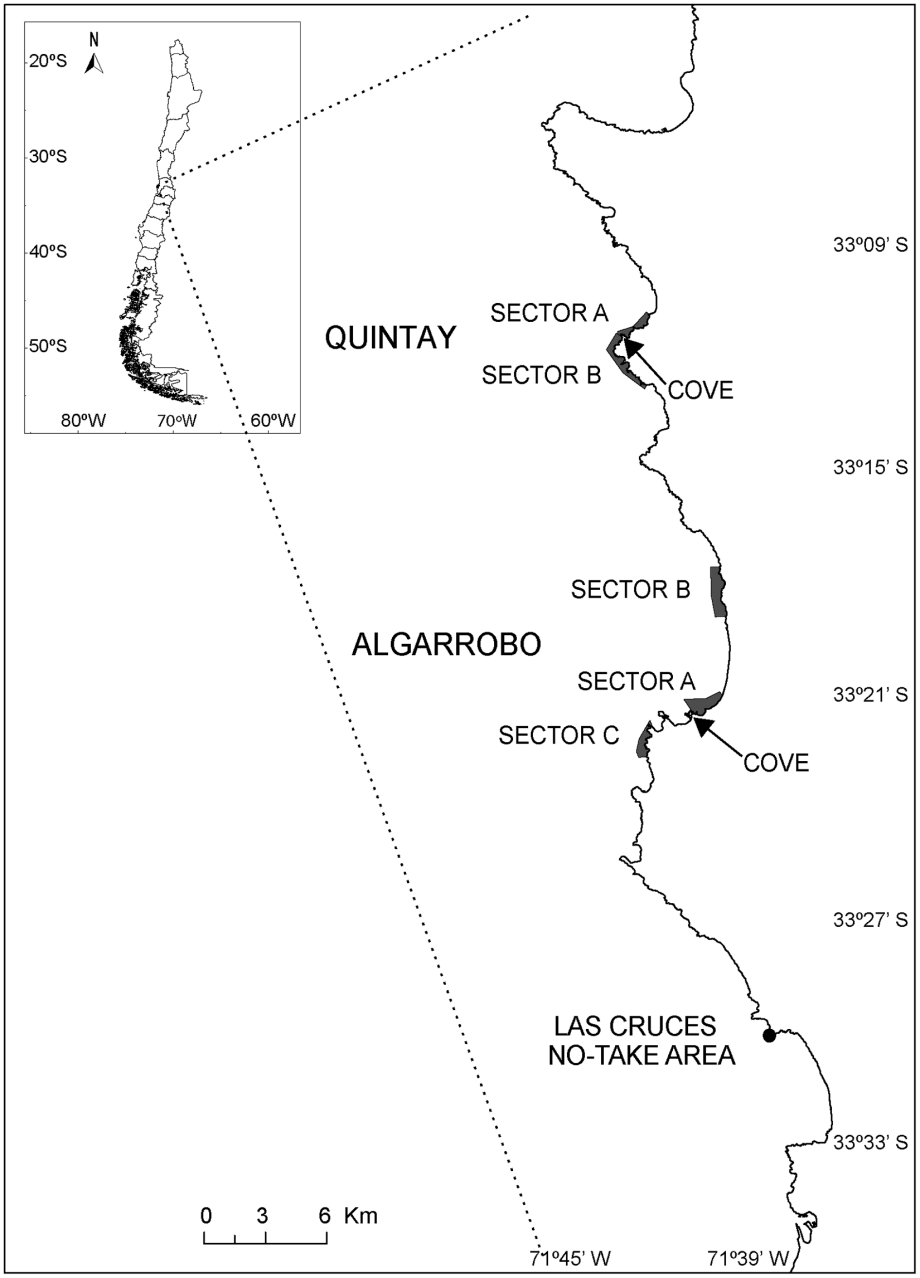
Map of the study area showing the no-take area of Las Cruces and different management areas where samples were collected. In the three sites (Algarrobo, Las Cruces and Quintay) traditional open access fishing grounds were also sampled.

This study was carried out in central Chile, between 33°11’ and 33°30’ S. In order to meet our goals, we selected two fishing coves or ‘Caletas’, Algarrobo and Quintay. Both study sites are long-established fishing coves in central Chile where a large fraction of pioneering studies on the influence of management regimes (MA) on benthic fisheries were conducted (e.g, [20,23,28]). The level of organization of the fishers into unions is similar in both Caletas, and both organizations are well structured [20,33]. A sign of the level of organization of the unions is how long fishers´ organizations have been able to maintain MAs; in both study sites MAs have been operating for over 20 years [34,35]. Fishers operate three independent MAs in Algarrobo (named here A, B and C) and two in Quintay (named here A and B), which are amongst the earliest officially implemented MAs in Chile (1999; Fig 1). It is important to point out that prior to the official implementation of the two management areas studied here, fishers unofficially established entry restrictions at first, and later as marine destinations [28,33]. Thus, our study does not encompass the recovery period of the stocks inside the MA. Clearly, fishers of both sites share a long experience managing TURFs, and have maintained the regular protocols established for MA (namely, conducting regular stock assessments, defining annual total allowable catches for each resource in conjunction with national authorities, enforcement protocols) for approximately 2 decades. In these studies, large samples of organisms of each target resource are obtained, and individuals measured (standard size measures for each species). Enforcement in MAs is conducted mainly by fishers, in coordination with local authorities (National Fisheries Service). Fishers organize surveillance protocols, by regularly patrolling along the coast to detect eventual poaching. The National Fisheries Service is in charge of enforcement in open access areas, but have very limited capabilities and only conduct occasional patrols [14,24].

At each site, we sampled all MAs and nearby OAAs (Fig 1). We considered as open access fishing grounds those fishing areas historically harvested by the fishers of each Caleta. Therefore, all open access fishing ground are nearby (but not necessarily adjacent) to the TURFs or NT area. In this paper we use MA when we refer specifically to our study sites, and TURFs when we refer to the territorial use rights system in general. We also included the single no-take (NT) area existing in the study region, Las Cruces (1 km of coastline) and sampled a similarly sized OAA nearby. The Las Cruces NT area showed comparable conditions to surrounding OAAs prior to being closed off from exploitation in 1982 [36], suggesting that this site was similar to the background areas, and therefore is a representative site in the study region. Also, significant increases in the biomass and sizes of exploited species have been reported in MAs since TURFs have been established, suggesting that the changes observed in the no-take area and MAs are related to changes in the management regime rather than to pre-existing conditions in the areas studied [28]. Site selection for TURFs seems to be mostly driven by the presence of historical fishing grounds in the vicinity to the cove rather than to clear variation in productivity (or habitat; [37]). In fact, fishers tend to protect MAs near to their fishing cove better due to the cost involved in patrolling [20]. Therefore, there is no clear evidence of special conditions within TURFs. Indirect evidence for similarity of environmental conditions in all fishing grounds comes from comparative studies examining body condition and investment in reproduction of keyhole limpets and sea urchins, which showed no differences between OAAs and protected areas (MAs and no-take areas; [38]). The total distance between the furthest north (Quintay) and furthest south location (Las Cruces) was 37 km, and the whole study area is located within the same oceanographic and ecological region [39]. Recent studies in the same study sites have shown that substrate composition and complexity did not differ between open access, TURFs and the no-take area of Las Cruces [20]. Therefore, it is reasonable to assume that habitat quality did not affect the collection of keyhole limpets and locos.

### Database

Catch curve analysis is a very old and simple approach, that focuses on the proportions of the different age and size classes harvested by a fishery, requires little information and fits both the comparative nature of our study, and the existing time series information on size distribution. We base the study in two sets of data. The first contains size frequency distribution data obtained in 2013, in MA, open access areas, and the no-take area in Las Cruces. The second contains size frequency distributions obtained only in MA, but annually between 1999 and 2013. The value of the first set of data is the comparative analysis of total mortality across management regimes to infer illegal fishing. The value of the latter is the possibility to relate the results of the snapshot estimate of total instantaneous mortality to the ranges normally observed in the study sites.

In order to compare the patterns of total mortality in fishing grounds under two management regimes, we conducted a survey in 2013 recording total length of locos (*Concholepas concholepas*) and keyhole limpets (*Fissurella* spp.) between January and May in the study sites (coves). To estimate the current instantaneous mortality rates of each target species, samples of both locos and keyhole limpets were collected in (a) 3 MAs in Algarrobo (different fishing grounds named here A, B and C), (b) 2 MAs in Quintay (A and B), (c) 5 historical fishing grounds in OAAs in Algarrobo (over approximately 10 km), and (d) 13 fishing grounds in OAAs in Quintay (over approximately 7 km). In addition, we sampled the no-take area in Las Cruces, as control area with no fishing impact (to estimate natural mortality) and one OAA fishing ground nearby. The selected OAA sampled areas correspond to the fishing grounds that are most frequently visited by fishers and there is no overlap in the fishing grounds between the study sites. Therefore, at each site we sampled several fishing grounds within the same level of protection (management regime). A total of 3 management regimes (no-take, MA, OAA), 3 sampling sites (Quintay, Algarrobo and Las Cruces) and 23 fishing grounds (besides the NT area) were sampled (Table 1).

**Table 1:** Sample size of locos (*Concholepas concholepas*) and keyhole limpets (*Fissurella* spp.) in the different management areas (MA) and open access areas (OAA) as well as one no-take area.

					*Concholepas concholepas*	*Fissurella* spp.
Site	Year	Management regime	MA	N° fishing grounds	Sample size	Sample size
**Las Cruces**	**2013**	**No-take**	**NA**	**1**	**282**	**352**
**Las Cruces**	**2013**	**OAA**	**NA**	**1**	**136**	**203**
**Algarrobo**	**2013**	**OAA**	**NA**	**5**	**422**	**724**
Algarrobo	1999	MA	A	-	1,062	2,266
Algarrobo	2000	MA	A	-	972	1,127
Algarrobo	2001	MA	A	-	595	281
Algarrobo	2002	MA	A	-	640	843
Algarrobo	2003	MA	A	-	210	211
Algarrobo	2004	MA	A	-	198	158
Algarrobo	2005	MA	A	-	143	156
Algarrobo	2006	MA	A	-	430	879
Algarrobo	2007	MA	A	-	995	1,044
Algarrobo	2008	MA	A	-	248	561
Algarrobo	2009	MA	A	-	252	196
Algarrobo	2010	MA	A	-	225	278
**Algarrobo**	**2013**	**MA**	**A**	**4**	**220**	**656**
Algarrobo	1999	MA	B	-	1,531	1,556
Algarrobo	2000	MA	B	-	991	1,590
Algarrobo	2001	MA	B	-	560	980
Algarrobo	2002	MA	B	-	656	1,350
Algarrobo	2003	MA	B	-	272	715
Algarrobo	2004	MA	B	-	325	499
Algarrobo	2005	MA	B	-	251	390
Algarrobo	2006	MA	B	-	635	728
Algarrobo	2007	MA	B	-	754	1,212
Algarrobo	2011	MA	B	-	349	363
**Algarrobo**	**2013**	**MA**	**B**	**5**	**296**	**424**
Algarrobo	1999	MA	C	-	1,207	1,182
Algarrobo	2000	MA	C	-	956	1,398
Algarrobo	2001	MA	C	-	1,075	1,294
Algarrobo	2002	MA	C	-	1,090	1,103
Algarrobo	2003	MA	C	-	1,289	589
Algarrobo	2004	MA	C	-	499	467
Algarrobo	2005	MA	C	-	209	169
Algarrobo	2006	MA	C	-	1,077	587
Algarrobo	2007	MA	C	-	878	467
Algarrobo	2008	MA	C	-	520	1,010
Algarrobo	2010	MA	C	-	175	73
Algarrobo	2011	MA	C	-	450	449
**Algarrobo**	**2013**	**MA**	**C**	**3**	**325**	**498**
**Quintay**	**2013**	**OAA**	**NA**	**13**	**1,352**	**2,699**
Quintay	2001	MA	A	-	6,753	6,561
Quintay	2002	MA	A	-	1,920	2,614
Quintay	2004	MA	A	-	443	1,040
Quintay	2006	MA	A	-	595	1,052
Quintay	2007	MA	A	-	1,083	1,602
Quintay	2011	MA	A	-	803	1,603
**Quintay**	**2013**	**MA**	**A**	**4**	**208**	**438**
Quintay	1999	MA	B	-	586	1,254
Quintay	2000	MA	B	-	4,906	1,775
Quintay	2001	MA	B	-	2,735	*
Quintay	2002	MA	B	-	2,506	2,486
Quintay	2004	MA	B	-	508	1,042
Quintay	2006	MA	B	-	446	1,593
Quintay	2008	MA	B	-	1,672	756
Quintay	2009	MA	B	-	478	1,378
Quintay	2011	MA	B	-	1,039	1,586
**Quintay**	**2013**	**MA**	**B**	**7**	**462**	**718**

In bold comparative samples taken in 2013. NA indicates that a given classification is not applicable.

*: Missing data. *Fissurella* species are not sorted by fishers, and the catch of these species are also pooled when reported in the fisheries statistics.

Fishers selected the different grounds (and fishing spots in each ground) where the fishing excursions were conducted both in MAs and OAAs. Different spots were sampled in each fishing trip. A small group of fishers were included in the study in each Caleta to minimize errors associated to the sampler. However, only fishers from each Caleta sampled within each site (cove), due to entry restrictions. Fishers did not receive a particular training for this study as they were expected to conduct a fisheries operation. It is important to remark that fishers from these Caletas have been collaborating with scientists conducting annual stock assessments in MA and are capable to follow scientific instructions. Fishers of all study sites have participated in collections to estimate abundance and size frequency distribution since the late 80s [28,40]. In our sampling in OAA and MA divers collected animals as part of their harvesting routine, selecting the starting point for each dive and covering as much area as possible in each sampling. All animals were collected through hookah diving. In all cases animals were returned to their habitat after length measurements were performed (see below). A fixed amount was paid for each fishing trip regardless of the number of dives or catch for the fishing operation. Due to restrictions related to the administration of the protected area, animals from the Las Cruces NT area were collected by scientists. In the NT scientists followed the same sampling protocol as carried out by fishers in MA, covering most of the NT area. Dives were performed from 2 to 20 meters depth and extended from 20 to 60 minutes (variable number of immersions). During that time divers covered highly variable areas (approximately 50 m around the boat), being limited by the time it took them to fill their mesh collection bags (“chinguillos”) with the resources.

The total length of each specimen was measured to the nearest mm with a Vernier caliper. Sample sizes ranged between 208 and 1,352 for *C*. *concholepas* and between 352 and 2,699 for *Fissurella* spp. in the different sites analyzed (see Table 1). Through a bootstrap resampling technique we estimated an n = 200 as the minimum sample size needed to obtain reliable size-frequency distributions for the target populations of the studied species. At Las Cruces OAA the number of locos collected by fishermen was low (n = 136). Abundances of benthic resources in the historical open access fishing grounds of Las Cruces have been lower than in other areas, due to exploitation and lack of compliance with regulations [28]. For this reason, it was not possible to conduct the catch curve analysis for neither locos nor keyhole limpets. The sample size for locos was smaller than our minimum sample size and the bi-modal frequency distribution for keyhole limpets was unsuitable for further analyses using this approach as employed in the remaining sampling sites.

In order to analyse the patterns of instantaneous total mortality in MAs, and thus evaluate if the snapshot estimates were consistent over time, we also obtained length data from annual stock assessments conducted during 14 years for each MA. The studies were conducted by an independent scientific team in collaboration with fishers, upon request of the fisheries administration. The same scientific team conducted the studies in both Caletas. The collection of animals for size measurement was carried out by artisanal fishers following the same procedure explained above. Since we applied the same protocol as used in previous studies, the sampling areas were selected by fishers and remained relatively constant across sampling trips (and between both groups of studies). Since the two study sites are among the first MAs established in Chile, we were able to compile data from 1999 to 2013. A total of 42,464 locos and 52,194 keyhole limpets were measured at the five studied TURFs between 1999 and 2013 (see Table 1). We do not expect to observe a pattern resembling an initial phase of recovery (lower exploitation rate) in total mortality because entry restrictions were imposed in both Caletas before the areas were officially declared [28,33].

### Ethics statement

The Chilean Navy and the Undersecretary of Fishing granted all necessary permission and permits to conduct the described fieldwork. Non-destructive manipulation of endangered or protected species was required. The study was carried out in strict accordance with the Guidelines for the Care and Use of Laboratory Animals (National Commission for Scientific and Technological Research, CONICYT, of Chile) and the “Bioethical and Biosecurity Committee” of the Faculty of Biological Sciences, Pontificia Universidad Católica of Chile (permit number CBB-233/2012). Special permits were obtained from the Administration of the Costal Marine Protected Area of Las Cruces.

### Data analysis

Size data were organized in 1-mm length intervals and lengths were transformed into ages using the von Bertalanffy growth model [41]. We used this model to allow comparison with previous mortality estimates which were also based on von Bertalanffy growth model both in *Fissurella* [42–44] and *Concholepas concholepas* [45–47]. Furthermore, when compared with the Gompertz model, claimed to be more suited to model invertebrate growth [Nash 1992 cited by 48], the von Bertalanffy model showed a better fit [44]. Age-frequency distributions, using 0.2-year size classes, were obtained for each site and exploitation regime by plotting the natural logarithm of the frequency as a function of the gastropod’s age. We used 0.2 age-classes because age specific mortality, and so the detection of the onset of the descending branch of the distribution may be masked using wider age-classes (please see explanation below). Then, fishing mortality was estimated from each age-frequency distribution by performing catch-curve analysis [49,50,51]. The catch curve analysis focuses on the slope of the descending section of the age-frequency distribution, which corresponds to the total instantaneous mortality (*Z*; a parameter in the regression, representing the sum of natural mortality and fishing mortality). Thus, we estimated *Z* through linear regression analysis, using the natural logarithm of the frequency at age as dependent variable and age as the independent variable (Table 2, Eq. 1). In addition to the assumptions of the catch curve model [51], we also assumed that (a) the growth parameter (*K*) is constant in both space and time (*K* = 0.433 for loco [46] and *K* = 0.160 for keyhole limpets [42,52]), (b) natural mortality is constant over the entire sampling region, and (c) a reliable estimate of natural mortality can be obtained by measuring total mortality in the no-take area of Las Cruces [25]. We are confident with these assumptions because previous studies in the same ecoregion [39], in exposed rocky shores, and using similar set of benthic species have shown no effect of site on relevant biological variables such as body condition and gonadosomatic index [38]. Moreover, no effect of upwelling was detected on relevant biological variables along the same study region (body condition and gonadosomatic index; [38]). Finally, the NT of Las Cruces is the best enforced marine protected area of Chile (although a minor level of poaching cannot be completely ruled out; [53]).

**Table 2:** Models used to evaluate the different hypotheses of this study, identifying the dependent and independent variables. The parameters of the regression are *Z* (slope) and B (intercept).

Catch curve-model					
Independent variable	Dependent variable (s)	Slope		Intercept	Equation 1
Age (A). Transformed from size data through the von Bertalanffy growth model	Logarithm of frequencies at age (log C)	Total instantaneous mortality (*Z*). *Z* = natural mortality (*M*) + fishing mortality (*F*)		Intercept (B) estimates the catch of age 0 animals would have been if they were as vulnerable to the fishery as fully recruited animals [Hilborn and Walters 1992]	Log C = A・*Z* + B
**Regional Patterns of Total Instantaneous Mortality among Levels of Human Impact**. H1 (loco): (a) *Z* is higher in MAs than in NT and OAAs; (b) Z is not significant different between NT and OAAs. H2 (limpets): *Z* in OAAs is higher than in MAs which in turn is higher than NT.					
Independent variable	Dependent variable (s)	Co-variable	Slope	Intercept	Equation 2
Management regime (MR). Fixed factor with three levels: NT (no-take), MA (management areas), and OAA (open access areas)	Logarithm of frequencies at age (log C)	Age (A)	*Z*	B	Log C = MR・*Z*・A + B
**Local Patterns of Total Instantaneous Mortality (among management regimes across sites)** H1 (loco): At both study sites *Z* is higher in MAs than in OAAs. H2 (limpets): At both sites Z in OAAs is higher than in MAs.					
Independent variable	Dependent variable (s)	Co-variable	Slope	Intercept	Equation 3
1. Management regime (MR). Fixed factor with two levels: MA (management areas), and OAA (open access areas) 2. Site (S). Fixed factor with two levels: Algarrobo y Quintay	Logarithm of frequencies at age (log C)	Age (A)	*Z*	B	Log C = S (MR)・*Z*・ A + B
**Local Patterns of Total Instantaneous Mortality (among management regimes within site)** H1 (loco): At each site *Z* is higher in MAs than in OAAs H2 (limpets): At each site *Z* in OAAs is higher than in MAs					
Independent variable	Dependent variable (s)	Co-variable	Slope	Intercept	Equation 4
Management regime (MR). Fixed factor with two levels: MA (management areas), and OAA (open access areas)	Logarithm of frequencies at age (log C)	Age (A)	*Z*	B	Log C = MR・*Z*・ Age + B
**Temporal Patterns of Total Instantaneous Mortality** H1 (loco and limpets): there are differences in Z of loco and limpets among years for each MA in the study region					
Independent variable	Dependent variable (s)	Co-variable	Slope	Intercept	Equation 5
Year (Y). Random factor with 15 levels, from year 1999 to 2013	Logarithm of frequencies at age (log C)	Age (A)	*Z*	B	Log C = Y・*Z*・Age + B

The catch-curve analysis requires defining: (a) how to deal with zero-catch age classes, usually located at the right-end of the curve, which might bias the estimation of the regression´s parameters, and (b) where the descending section of the catch-curve starts (i.e. which is the age of full recruitment to the fishery; [54]). In order to resolve the first issue all observations at the right-end of the age-frequency distribution that followed a gap of zero-catch for four or more age classes were excluded from further analyses. Regarding the second issue we fitted a continuous spline function to the discrete values of our catch-curves (recall that we use age-classes in the x-axis), and in each case we computed the maximum frequency age for the spline function to get the age of full recruitment to fishery. Although this is a straightforward method, the use of a continuous spline function to fit discrete data added unmeasured uncertainty to the setting of the onset of the descending branch of the age-frequency distribution plot. This may decrease the performance of subsequent linear models, particularly at the left hand of the plots (corresponding to smaller ages).

Subsequently, comparisons of slopes (*Z*) were conducted in order to analyze patterns from regional to local scales (see below). These comparisons were performed through analysis of co-variance (ANCOVA) using the natural logarithm of the frequency at ate as dependent variable, the factors of interest (e.g., management regime, site, time) as independent variables and age (of organisms in the catch) as co-variable. All the statistical analyses were carried out using the free software R version 3.2.3 [55].

#### a. Regional patterns of total instantaneous mortality among levels of human impact

We used a one-way ANCOVA to assess differences in total instantaneous mortality among the three levels of the factor management regime (NT, MA, and OAA) using age as a covariate (Table 2; E. 2). Due to large differences in the number of samples among OAAs between fishing sites (Algarrobo and Quintay), which could bias the regional estimate towards a particular site, a subsample of individuals was chosen from the area with larger sample size (Quintay). The selection of the subsample was done forcing its final frequency distribution to match the frequency distribution of the whole sample of age values for Quintay. Thus, the final subset of 422 loco individuals in Quintay was equivalent to the number of locos obtained at the OAAs in Algarrobo. Similarly, a subset of 724 keyhole limpets in Quintay matched the number of individuals measured in Algarrobo (Table 1).

#### b. Local patterns of total instantaneous mortality

In order to better understand variability between sites, between management regimes, and among MAs (since there are more than one MA per site), we scaled down the analyses to assess: (a) consistency of the patterns of fishing mortality, comparing instantaneous fishing mortality between management regimes within site, and (b) variability among MAs, within sites. In both cases, comparisons were made against the same set of OAA data. The Las Cruces site was excluded from this analysis as explained above. Therefore, at the local scale we compared only the two management regimes (MA and OAA). To test the consistency of management regime’s influence across sites, we conducted a two-way ANCOVA with factor Management Regime nested within Site in order to assess differences between each of the groups: Algarrobo OAA, Algarrobo MA, Quintay OAA and Quintay MA (Table 2; Eq. 3). Although previous studies did not show any effect of main environmental drivers (i.e. upwelling) on meaningful biological variables [38] along the study area, nested design was used to account for other local conditions that could affect each particular site. We pooled length data from all MAs (and OAA) within each site, obtaining a single set of observations for each level of management regime within sites. Then, *Z* was estimated. Additionally, to analyze patterns of mortality among MAs and OAA within each site, one-way ANCOVAs were performed (Table 2; Eq. 4). In our case, 3 MAs are in place in Algarrobo and 2 in Quintay. Since we sampled several historical fishing grounds, OAA were not paired with the MAs.

#### c. Temporal patterns of total instantaneous mortality

We conducted two statistical analyses in order to explore temporal patterns of total instantaneous mortality. First, following the same regional and local analysis than above, and in order to assess if the snapshot total instantaneous mortality rate for MA estimated in our comparative study was consistent over time, we compared (a) the regional average total instantaneous mortality over time (averaging *Z* across all MAs), using a one-way ANOVA and (b) local variability (within MA) in total instantaneous mortality across years using a one-way ANCOVA (Table 2; Eq. 5). Post-hoc tests were performed to identify which years were driving the significance of differences found in the main test. The R package “phia” was chosen for the post hoc analysis due to its flexibility in treating non-balanced data [56]. As explained above, actual enforcement started before official declaration of the TURF. Therefore, the 15 years of mortality estimates are not expected to reflect the recovery phase of the fishery after entry restrictions were imposed. We also compared the average total instantaneous mortality in MA in the study region (from 1999 to 2013) between the two species analyzed here using a Student’s t-test. Although the assumption of relatively constant recruitment (equilibrium conditions) is particularly critical in the comparison of catch curve models in marine invertebrates exhibiting complex life cycles, existing evidence showed that (a) recruitment of dominant marine invertebrates in the study area was remarkably persistent at mesoscales (kilometres, [57]), and (b) recruitment of dominant intertidal invertebrates (mussels, barnacles) were not altered even by large environmental sources of variability such as El Niño [58].

## Results

### Regional trends

**Fig 2:**
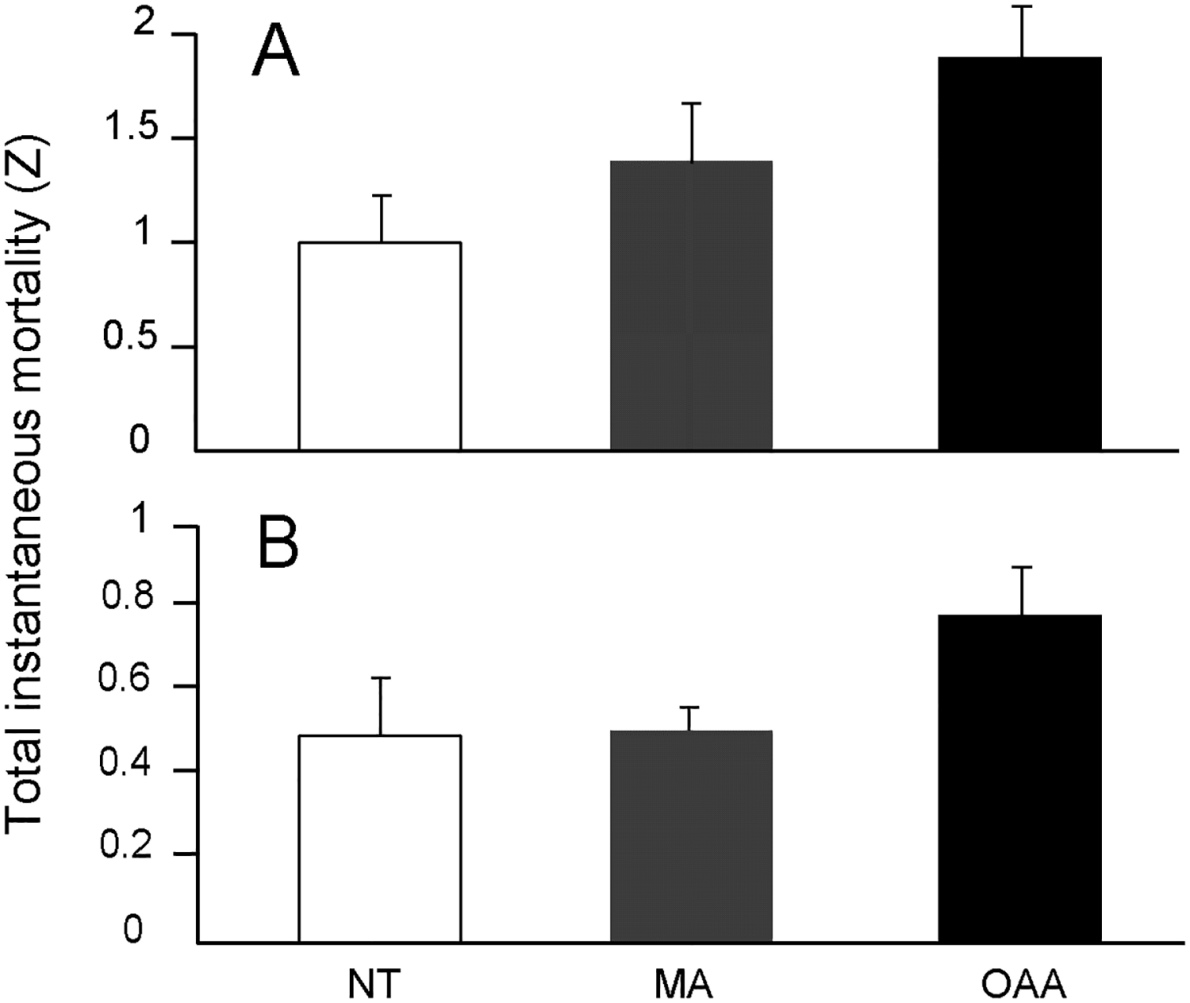
Regional trends in total instantaneous mortality (*Z*) in the three studied management regimes. (A) Loco (*Concholepas concholepas*). (B) Keyhole limpets (*Fissurella* spp.). NT: no-take area of Las Cruces; MA: Management Area; OAA: Open Access Areas. Error bars represent the 95% confidence intervals for the estimates of *Z*. Note the different scales in the y-axis between panels A and B.

Total regional instantaneous mortality of locos varied significantly among management regimes (F = 14.74, df = 2, 43, p < 0.0001) (Fig 2A). Post-hoc comparisons showed significant differences between: (a) OAAs and the NT from Las Cruces (F = 29.48, p < 0.001), (b) OAAs and MAs (F = 13.40, p = 0.001), and (c) MAs and the NT of Las Cruces (F = 5.86, p = 0.013). The steepest slope, indicating highest instantaneous fishing mortality, was detected in OAAs (*Z* = 1.88), followed by MAs (*Z* = 1.39) and NT (*Z* = 0.98). Thus, loco mortality in OAAs and MAs was 92% and 42% higher than in the NT area of Las Cruces, respectively.

Total regional instantaneous mortality of keyhole limpets also varied among management regimes (F = 14.35, df = 2, 91, p < 0.0001) (Fig 2B). Post-hoc tests revealed differences between OAAs and NT (F = 15.32, p < 0.001) and MAs and OAAs (F = 26.16, p < 0.001). However, no significant differences were observed between MAs and the NT area for these species (F = 0.76, p > 0.05). The highest mortality for keyhole limpets was estimated in OAAs (*Z* = 0.78), followed by MAs (*Z* = 0.49) and the NT from Las Cruces (*Z* = 0.48). Thus, regional keyhole limpet mortality showed a non-significant 2% increase from the NT area to MAs, and a significant 62% increase in mortality from the NT to the OAA.

### Local trends: Comparison of management regimes in the two study sites

**Fig 3:**
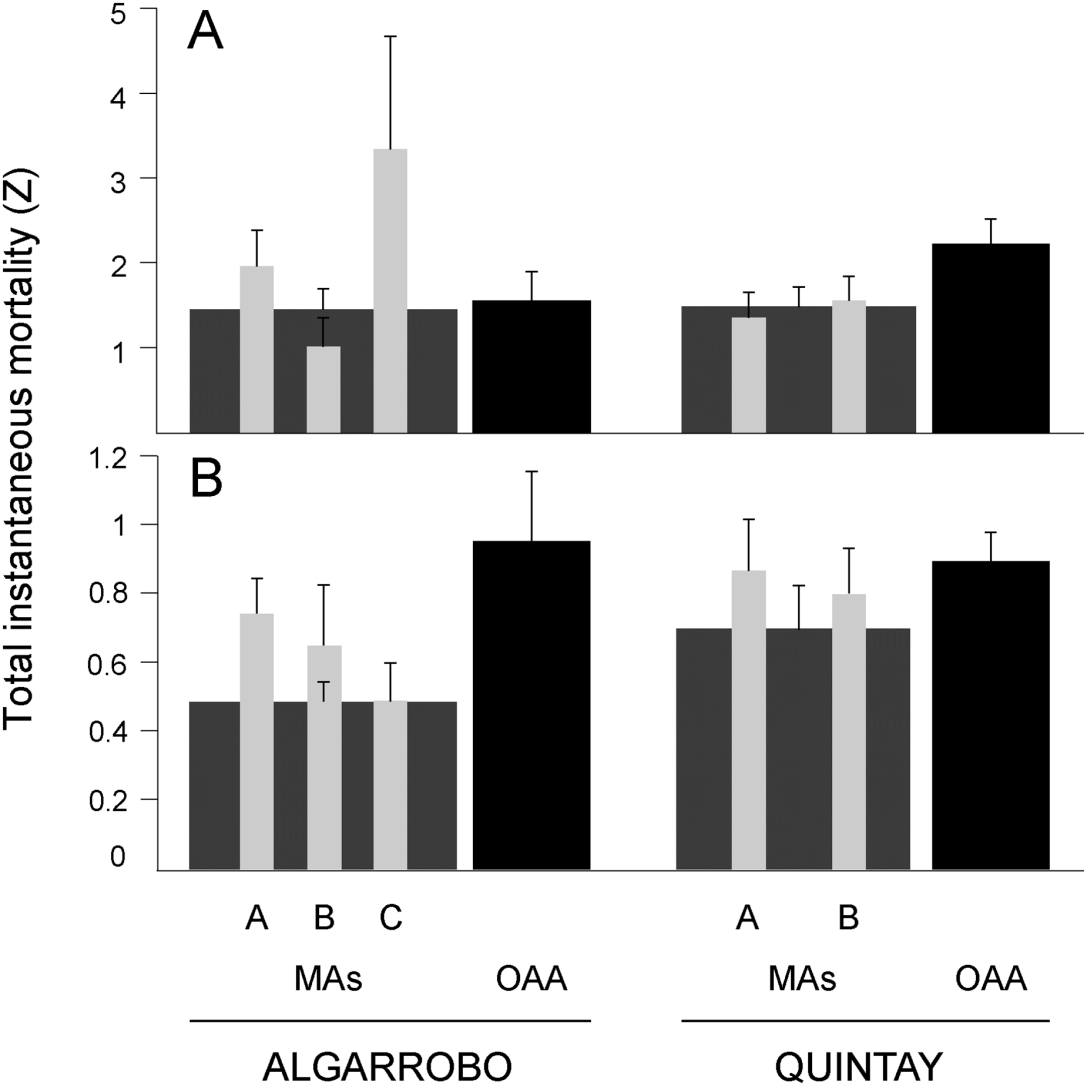
Local patterns of total instantaneous mortality (*Z*) in the two management regimes (MA and OAA) at the two study sites (Algarrobo and Quintay). (A) Loco (*Concholepas concholepas*). (B) Keyhole limpets (*Fissurella* spp.). Light-grey bars show *Z* for the different Management Areas (MA) within each site (A, B, C); dark-grey bars show *Z* estimates for the group of MAs of each site (data pooled for all MAs located within each site); black bars show *Z* in Open Access Areas (OAA). Error bars represent the 95% confidence intervals for the estimates of *Z*. Note the different scales in the y-axis between panels A and B.

The clear regional trend exhibited variability when we scaled down the analysis separating sites (Algarrobo and Quintay). Differences in total instantaneous mortality between management regimes were not consistent between sites, neither for locos (ANCOVA, Site x Management Regime interaction: F = 5.13, df = 3, 53, p = 0.003; Fig 3A), nor keyhole limpets (ANCOVA, Site x Management Regime interaction: F = 14.41, df = 3, 110, p < 0.001; Fig 3B). Post-hoc comparisons showed that in Quintay total instantaneous mortality was greater in OAAs than in MAs for locos (F = 11.47, p = 0.001) but not for keyhole limpets (F = 1.17, p = 0.28). Contrastingly, in Algarrobo no difference in total instantaneous mortality was observed between OAA and MA (F = 0.35, p = 0.556) for locos, but higher total instantaneous mortality was observed in OAAs than in MAs for keyhole limpets (F = 21.01, p < 0.001).

Scaling down one more step in the analysis, to compare each MA separately, along with adjacent OAAs in that site, we found that total instantaneous mortality of loco (Fig 3A) differed among the different MA and OAA, both in Algarrobo (ANCOVA: F = 7.34, df = 3, 40, p < 0.001) and Quintay (ANCOVA: F = 8.048, df = 2, 32, p = 0.0013). Total instantaneous mortality of loco in MA C from Algarrobo (*Z* = 3.27) showed the highest snapshot estimation, almost doubling any other estimates from Algarrobo, including the OAAs. In Quintay the highest total instantaneous mortality was found in the OAAs (*Z* = 2.18). No difference in loco mortality was found between the two MAs. Total instantaneous mortality of keyhole limpets (Fig 3B) also showed significant differences among MAs and OAA in Algarrobo (ANCOVA: F = 7.40, df = 3, 84, p < 0.001) but not in Quintay (ANCOVA: F = 0.53, df = 2, 73, p = 0.59). In Algarrobo, the post hoc tests revealed significant differences between MA A and C (F = 10.06, p = 0.01) and between Sector C and the OAA (F = 19.24, p < 0.001). The highest total instantaneous mortality of keyhole limpets in Algarrobo was observed in MA A and OAA (*Z* = 0.74 and 0.96, respectively). In Quintay, total instantaneous mortality estimates ranged between 0.80 and 0.90.

### Historical trends in co-managed areas

**Fig 4:**
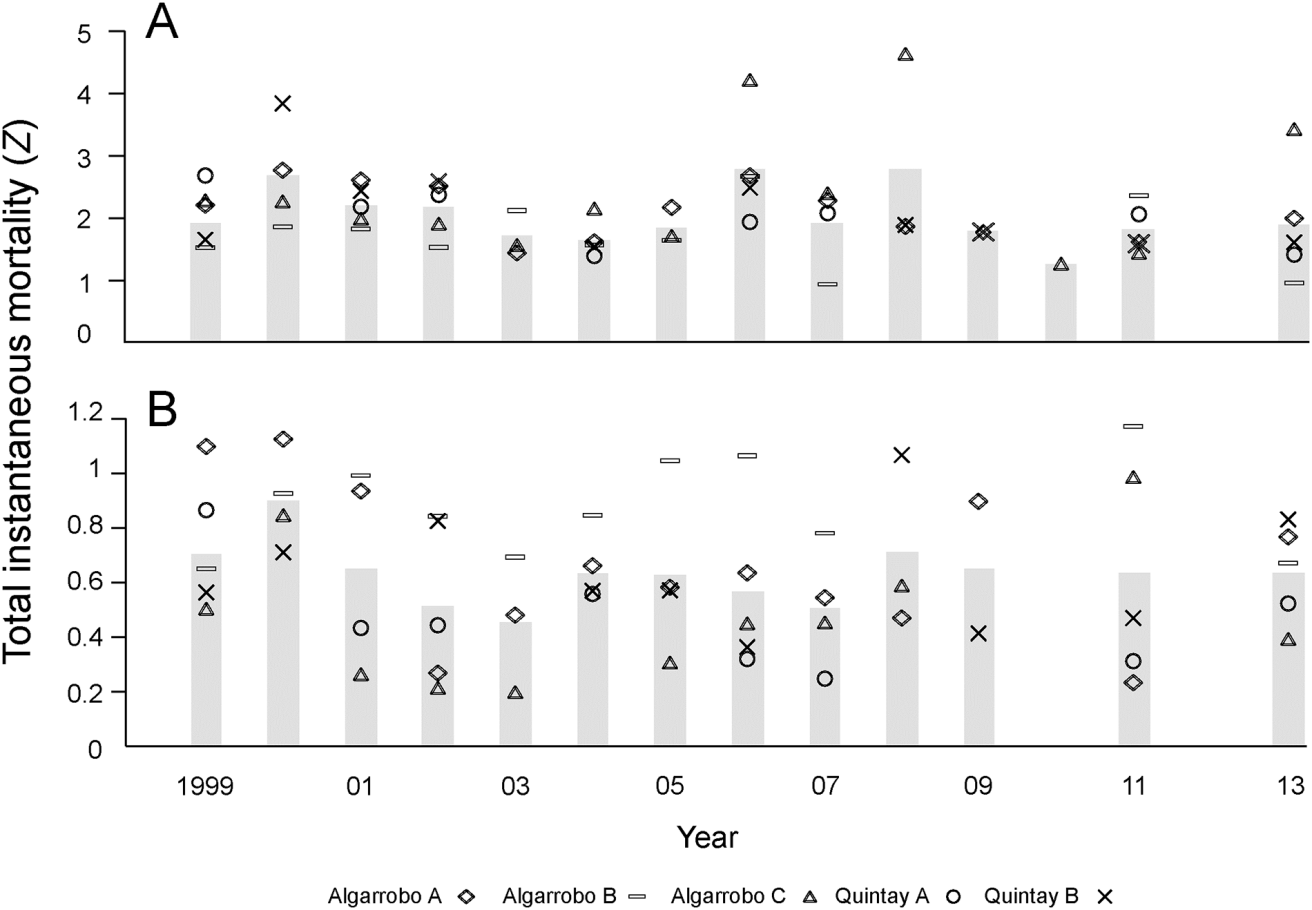
Historical trends in total instantaneous mortality (*Z*) over a 14-year period, at five management areas located in two sites of central Chile: Algarrobo and Quintay. (A) Loco (*Concholepas concholepas*). (B) Keyhole limpets (*Fissurella* spp.). Grey bars represent the average total instantaneous mortality (across sites). Letters A, B or C after the name of a site indicate individual Management Areas. Note the different scales in the y-axis between panels A and B.

At the regional level, average total instantaneous mortality across MAs did not show significant differences among years neither for locos (ANOVA: F = 0.93, df = 1, 52, p = 0.34; Fig 4A) nor for keyhole limpets (ANOVA: F = 0.31, df = 1, 51, p = 0.58; Fig 4B). On average, total instantaneous mortality of locos during the 14 years analyzed here was 1.99 (SE = 0.09), particularly influenced by total mortality between 1999 and 2007. Total instantaneous mortality of keyhole limpets between 1999 and 2011 was 0.60 (SE = 0.03), ranging between 1.14 (at Algarrobo B in 2011) and 0.19 (at Algarrobo C in 2003).

As for the snapshot analysis, the clear regional trend in total instantaneous mortality exhibited large variability when we scaled down the analysis to the local level (separating MAs). Total instantaneous mortality of locos and keyhole limpets varied significantly among years at each of the studied MAs (ANCOVAs; always p < 0.01). In Algarrobo higher mortality was observed mostly in the first 3 years of the time-series for both resources. However, for loco, the most conspicuous differences were found in Algarrobo C for the years 2006 and 2008 (p < 0.01) and for Quintay B in 2000 (p < 0.01). For keyhole limpets, differences among years at the different MAs were more erratic, although instantaneous mortality rates in Algarrobo B was always higher than the average (see below).

Total instantaneous mortality of keyhole limpets, on average across years, was significantly lower than mortality of locos in MAs (Student-t test = 13.93, df = 105, p < 0.0001). It is important to note that in certain years (2000, 2006, 2008), the instantaneous mortality of locos from particular areas (Algarrobo MA C and Quintay MA B) doubled the average annual mortality (Fig 4A). Similarly, local mortality rates double the annual average were observed for keyhole limpets (Fig 4B).

## Discussion

Using the scarce information available for artisanal benthic fisheries in central Chile, we were able to apply a very simple method, catch curve analysis, to explore patterns of fishing mortality. We recognize that the approach used for our analysis has several limitations and is sensitive to violations of underlying assumptions [54,59]. However, under rigorous examination catch curve analyses can be used. In fact, the potential of catch curve analysis has recently been highlighted for data-limited situations [60,61], which comprise a large fraction of exploited fisheries worldwide [3]. Thus, catch curves, along with other data-limited assessment methods, are potential tools for studying the large number of unassessed, data-poor fisheries around the world and providing valuable information for fisheries management [62–65]. Here we used this classical but simple approach to infer illegal fishing. Clearly, none of these alternative approaches substitute the urgent need for formal direct assessment of artisanal fisheries worldwide, or more powerful methods to determine illegal fishing, when data become available.

Our estimates of total and natural mortality (inside the NT area) of locos falls within the ranges of previous studies [28,40,45,66], providing support to our analysis. We were able to obtain basic indicators for the degree of fishing pressure across a range of human impact (or protection level), which provides evidence for the influence of protection in reducing fishing mortality. Our results also allow us to infer high levels of illegal fishing of the most valuable resource (locos) in OAAs, and suggest the overall effectiveness of TURFs to decrease fishing mortality in recent times. Nevertheless, the effectiveness of each MA needs to be evaluated on a case by case basis since the patterns of total mortality were not consistent over space and time, and sporadically very high fishing mortality was observed inside TURFs.

Our analysis of mortality patterns under different management regimes yielded important results for evaluating and comparing the protection provided by partially and fully protected areas. The catch curve analysis showed mortality patterns that overall correlated with entry restriction to fisheries. Our results show that the NT exhibits the lowest total mortality, suggesting that the no-take area of Las Cruces confers protection to exploited resources, although we cannot completely disregard poaching (the NT of Las Cruces is the best enforced marine protected area of Chile, according to Navarrete et al. [53]). We notice that our estimates of fishing mortality in MAs is 44% higher than official estimates using similar approaches [*F* = 0.27; 67]. Although we cannot disregard a potential influence of the methods used for estimating fishing mortality, our interpretation of the higher fishing mortality in MAs of our study stems from the differences in the spatial scales of the analyses. Our study was conducted along the most populated fraction of the coastal region of Chile (Region of Valparaíso), where higher fishing impact is expected, while official estimates average fishing mortality nationwide (including also very isolated areas). Our results also highlight the value of partially protected areas, such as TURFs, in general. Regionally, the effect of entry restrictions was particularly relevant for the most valuable and exploited resource: the loco. Loco mortality in the NT was 92% lower than in OAAs, and 42% lower than in MAs. Therefore, MAs provide some level of protection for locos. A similar trend was observed for keyhole limpets between OAAs and the NT. However, MAs seem to reach a level of protection for keyhole limpets similar to the NT, highlighting the potential of TURFs for the conservation of some resources [20]. Low fishing mortality in MAs is expected to occur, first, because entry regulations associated to TURFs lower fishing effort, and secondly because fishers do not always harvest the total allowable catch in MAs, particularly of the less valuable resources. The overall conclusion, based on our regional estimates, is that partially protected areas such as TURFs can provide ancillary benefits for marine conservation, substantially lowering total mortality of ecologically and economically important species despite the fact that local analysis showed strong variability in instantaneous total mortality among MAs and over years. The patterns of abundance of exploited resources reported across the NT area of Las Cruces, MA and OAA [20] agree with our estimates of mortality. Recent studies have also shown that meaningful biological variables such as density, biodiversity, and mean size in MAs are similar to those from no-take areas and higher than in OAAs, suggesting additional benefits of TURFs for marine conservation [20]. The role of TURFs in marine conservation is particularly relevant in central Chile, where socio-economical resistance to implementing NT areas occurs due to conflicting interests between fisheries and conservation needs [26]. However, the overall value of TURFs for marine conservation needs to be analyzed carefully. Our results suggest that a case-by-case examination of MAs is required considering the variability in total mortality of the main resources between MAs and over time.

In addition to the expected overall trend showing greater total mortality as levels of human impact (fishing) increase, our results also highlight (a) large and consistent differences in total mortality between locos and keyhole limpets, (b) contrasting patterns of total mortality in management areas over time (with sporadic peaks), and (c) large variability among MAs at relatively small spatial scales. The difference in total instantaneous mortality between the two resources may, at least in part, stem from natural differences in natural mortality between species (loco mortality was double that of keyhole limpets under no fishing conditions). However, the observed difference in natural mortality between both species by itself cannot explain the differences in total instantaneous mortality rates observed between the NT and exploited areas. We do not expect that violations to the constant recruitment or constant natural mortality assumptions of the model could affect differentially the two gastropod species as to generate total instantaneous mortality of locos between 2.3 and 2.8 higher than for keyhole limpets between NT and exploited areas. Both species exhibit a larval phase transported by coastal currents to similar recruitment habitats [38]. We hypothesize that fishers’ preference for the most traditional and valuable resource may determine the difference in total mortality between species in exploited areas.

Total instantaneous mortality estimated in MAs in our snapshot analysis fell within the range of total mortality estimated over the last 13 years for both species, suggesting that patterns of mortality showed variability but have not changed in a systematic fashion over time. Moreover, it is remarkable that the pattern of total mortality among MAs in our snapshot estimate mimics the pattern of average total mortality for the time series (average across years for Algarrobo A = 2.03; Algarrobo B = 1.67; Algarrobo C = 2.3; Quintay A = 1.81 and Quintay B 2.03). Thus, our results show that the current (snapshot study) and temporal levels of total instantaneous mortality (natural and fishing mortality) for locos in some of the MAs considered in this analysis (which are among the model MAs of Chile heralded as management and conservation success stories) are relatively close to the mortality levels reported shortly before overexploitation of locos in the late 80s (*Z* = 1.8; average mortality across time for MAs was 1.99) [28,40,45,66]. Thus, our results also suggest that not all MA are equally effective in the protection they provide for the loco. The levels of mortality from the late 80s, which were similar to what was observed on average in OAAs in our study (and sporadically in some MAs), drove to a total fishing ban after signs of overexploitation were observed [22]. Fishing mortality of keyhole limpets seems to be low in MAs, assuming there is no poaching in the NT area, but it is two times higher in OAAs. The current fishing mortality of keyhole limpets in OAAs is similar to recent official estimates for MA (*F* = 0.27) [67].

In contrast to the consistent regional patterns between species across levels of human impact, site-to-site variability was observed for both resources in MAs. Some alternative hypotheses that may explain this pattern are: (a) variability in natural mortality creating bias in the estimate of total mortality (violation of constant mortality assumption), (b) variability in recruitment creating bias in the estimate of total mortality (violation of the constant recruitment assumption), and (c) differences in fishers’ behavior towards the MA resulting in differences in surveillance and enforcement intensity. We cannot completely rule out the problems associated to violations of the assumptions of the model. Differences in natural mortality among sites might occur because of local environmental and ecological conditions. However, several pieces of evidences suggesting that across the study sites, no differences in meaningful biological variables (body condition, reproductive output), were observed [38]. There is also evidence of temporal and spatial persistence in recruitment in the study area [57, 58]. And finally, a clear effect of management regime on several biological variables (density, size, biomass, species richness) was reported for the study sites [20]. Based on this evidence, the most plausible hypothesis to explain patterns of mortality is differences in fishing effort between management regimes but also among MAs. We highlight the potential influence of differences in surveillance levels assigned among MAs, even those administered by the same group of fishers. Surveillance implies costs that must be paid by fishers in the co-management system associated to TURFs. So, if the net economic productivity of a TURF is reduced, then surveillance decreases, and the TURF may even be abandoned [68]. Surveillance tends to be more efficient in the MAs that are closer to the cove, and driven by the most valuable resource [28,69]. In Quintay, both MAs are at similar distances to the cove, and no difference in total instantaneous mortality was observed for locos and keyhole limpets between them. In contrast, in Algarrobo total instantaneous mortality seems to be related to the distance from the cove (and therefore, with degree of surveillance). The best enforced area is sector A, which is closest to the cove (Fig 1). The most valuable resource exhibits the lowest mortality in sectors A and B. Sector B, in fact, is located farthest away from the cove, but it is naturally protected by environmental conditions (waves) and lack of roads that restrict access. Sector C can be easily accessed by fishers. Therefore, it may be more vulnerable to poaching, which might explain the highest mortality of the most valuable resource. It is important to test this hypothesis in the future, because illegal fishing may take place in MAs [9]. The critical effect of surveillance in co-managed areas has already been described by many authors for other regions of the world (e.g. [70,71]).

The general pattern of higher mortality in OAAs in comparison to fishing-restricted areas fits our predictions for keyhole limpets. However, our results suggest illegal fishing of locos in OAAs, since the loco fishery is partially closed (open in MA, closed in OAA). Total mortality of locos was higher in OAAs than in MAs, despite loco fishing being banned in OAAs since 1993 [19,20,23]. Based on our estimate of natural mortality, which is similar to other studies [28,40,45,66], we show that total mortality in OAAs doubles our estimate of natural mortality from the NT. If we assume that the patterns of mortality reported here across management regimes reflect illegal fishing in OAAs, and roughly extrapolate our finding to the entire administrative region of central Chile where the study was conducted (Valparaíso), illegal fishing may equal legal catch. About 10 years ago a similar level of illegal fishing was estimated nationwide by other methods [9]. We acknowledge that our estimate of illegal fishing of locos is only based on spatial exploitation bans, and from this result we suggest the potential of the approach to infer illegal fishing under special conditions (contrasting management regimes and bans). However, the impact of illegal fishing may be higher and affect more species if other regulations such as minimum legal size or reproductive bans were included in the analysis. The comparative approach used here allows inferring illegal fishing exclusively on OAAs estimates, however, poaching has been reported in TURFs [9,31] and may have even increased over time. As highly productive historical fishing grounds in OAAs become depleted by illegal fishing, the fishing grounds contained within co-managed areas [72] will then attract poaching. A systemic lack of enforcement is clearly the largest problem associated to the lack of effectiveness of fishing regulations in Chile [14,24]. Therefore, the impact of illegal fishing on this data-poor artisanal fishery may have far reaching consequences on the sustainability of benthic resources. Our results suggest the need for a revision of the effectiveness of management regulations in this artisanal fishery, since illegal fishing threatens integrity and health of ecosystems, as well as the socio-economic well-being of the people that depend on these resources [73,74]. Existing evidence [20], linked to our results, highlight the urgent need to analyze the enforcement system in place and the value of an ineffective permanent ban on locos. In our particular system, illegal fishing may also affect the integrity of TURFs, as their persistence is highly dependent on healthy fishing in OAAs since local populations are connected by larval stages [69,75].

Although the overall status of artisanal fisheries has greatly been improved with the implementation of bottom-up co-management policies (TURFs; [29,30]), we should not become overoptimistic. The actual high levels of illegal fishing in OAAs inferred from this study suggest that the overall status of the fishery might not be as promising as the patchy assessments in TURFs have shown [23]. The landscape patterns seem to be more relevant than the mosaic of healthy TURFs, considering the spatial scales of the processes regulating the dynamics of these resources [32,72] and the strong dependency of fishers on OAAs. Fishing locos in co-managed areas is sporadic and generates only a small additional income to fisher unions, which the members divide-up once or twice a year [12]. In addition, the high economic costs of maintaining co-managed areas (mainly annual stock assessments and surveillance, as explained above) may render them unsustainable as a sole source of income [68], driving exploitation of OAAs. The high commercial value of loco prompts fishers to poach outside their co-managed fishing grounds in an attempt to offset their economic loss [76]. Thus, the health of TURFs largely depends on overexploitation in OAA. The key to the successful management of loco, and surely for several other marine resources, involves novel solutions and adaptive legislation that allow regulations to be modified as management scenarios evolve [77]. Generating pertinent data that will permit a profound analysis and assessment of these fisheries, and artisanal fisheries in general, will be paramount to achieving these goals. We highlight the value of fishery independent monitoring, such as the one performed in this study, to understand the health of a closed fishery.

In our opinion, the analysis comparing instantaneous fishing mortality across management regime suggests that catch curve analysis could be used for other explorations of the impact of regulations. First, it could be applied to other unassessed species, or geographic regions, to determine and compare exploitation levels under different management regimes and, consequently, the extent of illegal fishing. Second, it might also contribute to our understanding of the interplay between two contrasting management strategies in determining the overall status of exploited stocks whose dynamics do not match the scale of management [24, 72, 78]. Currently there is an overemphasis on TURFs assessments and little attention has been paid to OAAs [79] despite the fact that a single stock is exploited in both types of areas. Finally, it is also interesting to apply this method to explore the extent to which the “abundance” of TURFs may drive overexploitation (and illegal fishing) in OAAs due to effort displacement. The number of TURFs in Chile has increased substantially and we know very little about the impact of TURFs on open access areas as a result of effort displacement.
