# Correction: Exploitation rates of two benthic resources across management regimes in central Chile: Evidence of illegal fishing in artisanal fisheries operating in open access areas

**DOI:** 10.1371/journal.pone.0191166

**Published:** 2018-01-09

**Authors:** Miguel Andreu-Cazenave, Maria Dulce Subida, Miriam Fernandez

Reference 49 contains two individual references. The correct reference 49 is: Beverton RJH, Holt SJ. On the dynamics of exploited fish populations. London: Her Majesty’s Stationary Office. 1957. The correct reference 50 is: Hilborn R, Walters CJ. Quantitative fisheries stock assessment and management. Chapman-Hall: New York. 1992. All subsequent references should be shifted up by one.

Reference 79 is incorrectly included in the article. All of the in-text citations appear correctly throughout the paper.
